# Development and comparative study of chemosynthesized antigen and mimotope-based immunoassays for class-specific analysis of *O,O*-dimethyl organophosphorus pesticides

**DOI:** 10.1038/srep37640

**Published:** 2016-11-22

**Authors:** Fengchun Zhao, Huimin Wang, Xiao Han, Zhengyou Yang

**Affiliations:** 1 Department of Microbiology, College of Life Science, Key Laboratory for Agriculture Microbiology, Shandong Agricultural University, Taian 271018, China

## Abstract

The multi-residue determination of organophosphorus pesticides (OPs) is an important task due to the wide application and high toxicity of OPs. However, there is no promising immunoassay to monitor the multi-residue of *O,O*-dimethyl OPs. In this study, a monoclonal antibody (mAb) against a generic hapten of *O,O*-dimethyl OPs (*O,O-dimethyl O-*(*3-carboxyphenyl*)*phosphorothioate*) was prepared. To develop an effective class-specific immunoassay, two strategies were performed to select the appropriate coating antigen or competing antigen. On the one hand, a total of 20 haptens were chemosynthesized, attached to ovalbumin for use as coating antigen candidates, and selected by direct competitive ELISA (dcELISA). As a second strategy, mimotopes of the mAb were selected from a random phage-display peptide library by panning, and the optimum mimotope was expressed as a fusion protein and biotinylated *in vitro*. Based on the selected chemosynthesized coating antigen and the biotinylated mimotope fusion protein, two sensitive broad-specificity dcELISAs were developed. The sensitivity, selectivity and practicability of the two immunoassays were compared. The results demonstrated that both methods showed similar selectivity and sensitivity and were reliable for *O,O*-dimethyl OP residues screening. However, the screening operation of mimotopes was much simpler and safer compared to the preparation of chemosynthesized coating antigens.

Pesticides play an important role in improving the quantity and quality of crops^1^. However, with their increasingly wide application, public concern regarding pesticide residues contaminating of food and environment has risen due to the high toxicity and relative stability of pesticides^2^,^3^. Organophosphorus pesticides (OPs) are highly toxic pesticides that have broad-spectrum insecticidal activity and relatively low stability and have been broadly reported in food and environmental samples^4^,^5^. Thus, OP residues monitoring has great significance not only for the protection of human health but also for international trade and regulatory control^6^.

The broad-specificity immunoassays have been used to determine a group of pesticides which have a common structure. The advantage of this kind of immunoassay is that if the total quantity of pesticides in a sample is less than the maximum residue limit of the pesticides, we do not need further monitoring of these pesticides^7^. Most OPs share a common structure: *O,O*-diethyl or *O,O*-dimethyl phosphorothioate. Broad-selective immunoassays for *O,O*-diethyl OPs have been successfully developed based on the common structure (*O,O*-diethyl phosphorothioate), and more than 10 *O,O*-diethyl OPs could be detected with high sensitivity^7^,^8^. However, for *O,O*-dimethyl OPs, the developed broad-selective immunoassays indicated that less than 5 *O,O*-dimethyl OPs could be identified with high sensitivity^9^,^10^,^11^,^12^. Therefore, a new immunoassay with broader selectivity and higher sensitivity for *O,O*-dimethyl OPs is necessary.

The sensitivity of an immunoassay for low molecular weight compound is based on not only a superior antibody but also the quality of antigen^13^. The sensitivity of the immunoassay could be significantly improved by using an appropriate variant of the immunized hapten as a competitor^14^,^15^,^16^. For traditional optimization of antigens, researchers usually prepared a series of compounds using chemical synthetic method and coupling them to carrier protein for use as candidates. The optimum antigen was selected by immunoassays^7^,^17^ or molecular modeling^16^,^18^. These strategies were proved to be feasible in previous studies, but the preparation of candidate haptens was always “trial or error” and required considerable effort in chemical synthesis^16^,^19^. In addition, the high toxicity of some haptens posed a threat to the environment and human health^12^,^19^. Since the peptide mimotope-based immunoassay was successfully developed for low molecular weight compound (deoxynivalenol)^20^, an increasing number of researchers have begun to select environmentally friendly phage-display peptides (mimotopes), which can mimic the antibody binding site, to replace the chemically synthesized conjugates^12^,^19^,^20^,^21^,^22^,^23^,^24^,^25^,^26^. The immunoassays based on mimotopes, used as peptide-phage^12^,^21^,^22^,^23^, peptide-MBP fusion protein^19^,^24^ or synthesized peptide forms^25^,^26^, have shown their advantage in low molecular weight compound detection. In this study, a monoclonal antibody (mAb) with extraordinary broad detection spectrum for *O,O*-dimethyl OPs was produced. Additionally, two sensitive immunoassays for *O,O*-dimethyl OPs based on chemosynthesized antigen and mimotope were developed, and the sensitivity, selectivity and practicability of the two immunoassays were compared.

## Results and Discussion

### Preparation of haptens

**Figure 1:**
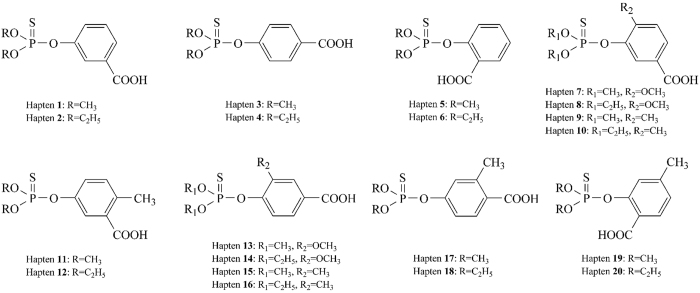
Structures of chemosynthesized haptens.

To develop an effective immunoassay for *O,O*-dimethyl OPs, 20 haptens were designed and chemosynthesized as candidates. All of the haptens contain a benzene ring with a carboxy group (carboxyl is used to couple hapten with carrier protein), whereas the differences among these haptens are the common structures of OPs (*O,O*-diethyl or *O,O*-dimethyl phosphorothioate), the position of carboxyl on the benzene ring, other substituents and their positions on the benzene ring. The structures of the haptens are shown in Fig. 1. Haptens **1–6** were prepared as described in our previous study^17^ and haptens **7–20** were newly synthesized and identified. The yields and ^1^H-NMR results of the haptens are shown in Supplementary Table S1, and the results indicate the successful of the haptens’ preparation. The synthetic route of hapten **1** is illustrated in Supplementary Fig. S1. The three-step synthetic method employed in this study provided the high purity and yields of haptens without any complex operations.

### Preparation of immunogen and coating antigen candidates

Previous studies have demonstrated that broad-specificity immunoassays for *O,O*-diethyl OPs based on mAbs against hapten **4**^8^ and hapten **16**^7^ were successful, and the average 50% inhibition values (IC_50_) for 13 OPs were 103.2 ng/mL and 91.5 ng/mL, respectively. However, the mAb based immunoassay developed by Liu *et al*. for *O,O*-dimethyl OPs against a generic hapten (3-(4-dimethoxyphosphorothioyloxy phenyl)propanoic acid) showed that 6 *O,O*-dimethyl OPs could be identified with IC_50_ from 580 to 10470 ng/mL^10^. One of the reasons for the low sensitivity of the *O,O*-dimethyl OPs immunoassay could be attributed to the long spacer arm between the aromatic ring and the carboxy group of the immunized hapten, which resulted in a significant recognition of the spacer arm during antibody formation^8^. The other reason might be that the substituent position on the benzene ring also had an obvious effect on the selectivity of the developed broad-specific antibodies^7^. Our previous study demonstrated that the mAb against hapten **2** (owning *O,O*-diethyl phosphorothioate with the carboxy group on the meta position of the benzene ring) exhibited broad selectivity to not only *O,O*-diethyl OPs but also *O,O*-dimethyl OPs^17^. This enlighten us that the carboxyl group at the meta position on the benzene ring may be helpful for promoting the broad-selectivity of the immunoassay for *O,O*-dimethyl OPs. Therefore, hapten **1** which possesses the generic structure of *O,O*-dimethyl OPs (*O,O*-dimethyl phosphorothioate) and was linked to the carboxyl group through the benzene ring in the meta position was selected as immunized hapten and covalently attached to BSA for use as immunogen.

Additionally, an appropriate coating antigen is always necessary to improve the sensitivity of the immunoassay for low molecular weight contaminants^7^,^16^. Thus, all of the 20 haptens were covalently attached to OVA with the active ester method and used as coating antigen candidates in the following study.

### Preparation of monoclonal antibodies

After three times immunization with hapten **1**-BSA, noncompetitive indirect ELISAs based on hapten **1**-OVA (homologous coating antigen) and hapten **5**-OVA (heterologous coating antigen) were performed to determine the titer of the antisera. Mouse 5, which showed the highest titer for both homologous and heterologous coating antigens (Supplementary Fig. S2a and S2b), was selected and euthanized for cell fusion. The competitive indirect ELISA (ciELISA) results demonstrated that the antiserum of Mouse 5 showed higher sensitivity to parathion-methyl when heterologous coating antigen was used (Supplementary Fig. S2c). Therefore, hapten **5**-OVA was used for the following hybridoma cell screening. After cell fusion, the culture supernatants of the hybridomas were screened by the heterologous-ciELISA, and two mAbs that showed high sensitivity to *O,O*-dimethyl OPs were obtained (named mAb3C9 and mAb4D11, respectively). The mAb3C9 was identified as an IgM with λ light chain, and the mAb4D11 was identified as an IgG1 with κ light chain. Both mAbs were labeled with horse radish peroxidase (HRP) using a modified NaIO_4_ method and used to develop direct competitive ELISA (dcELISA) in the following study.

### Development of chemosynthesized antigen-based dcELISA

To select the optimum coating antigens for the two mAbs, all of the 20 coating antigens were tested for plate coating using parathion-methyl as analyte. The maximal absorbance (*A*_max_), IC_50_ values of parathion-methyl and ratio of *A*_max_/IC_50_ were determined by dcELISAs and the results are shown in Table 1. MAb3C9 showed low sensitivity to parathion-methyl when haptens possessed of the generic structure *O,O*-dimethyl phosphorothioate, and showed high sensitivity when haptens possessed of the generic structure *O,O*-diethyl phosphorothioate (IC_50_ value: hapten **1 **> hapten **2**, hapten **3 **> hapten **4**, hapten **5 **> hapten **6**, and so on). Hapten **6**-OVA, which resulted in the highest ratio of *A*_max_/IC_50_ of parathion-methyl, was selected as the optimum coating antigen for mAb3C9. In contrast, for mAb4D11, the mAb showed higher sensitivity to parathion-methyl when haptens possessed of the generic structure *O,O*-dimethyl phosphorothioate; thus, hapten **7**-OVA, which exhibited the highest sensitivity was selected as the optimum coating antigen.

The sensitivity of ELISAs can also be affected by the ionic strength, pH and organic solvent concentration of the sample extract^27^. Therefore, these conditions of ELISA were also optimized. As shown in Supplementary Fig. S3, the ratio of *A*_max_/IC_50_ of parathion-methyl was used to estimate the influence on ELISA, and the highest ratio was selected as the optimized condition. For mAb3C9, high ionic strength (20 × PBS, 0.2 mol/L) could improve the affinity and sensitivity of mAb3C9, whereas low pH could improve affinity but decrease sensitivity. A high concentration of MeOH (>5%) led to serious antibody deactivation with no obvious improvement in sensitivity. Collectively, the optimal buffer solution used for mAb3C9 was 20 × PBS (pH 7.4) and 2% MeOH (Supplementary Fig. S3a). For mAb4D11, as illustrated in Supplementary Fig. S3b, the optimal buffer solution was 2 × PBS (pH 7.4), and the concentration of MeOH was 5%.

### Selectivity and sensitivity of chemosynthesized antigen-based dcELISAs

**Figure 2:**
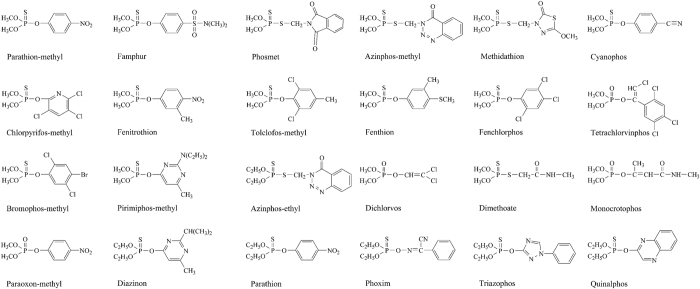
Structures of organophosphorus pesticides.

The structures of the 24 kinds of OPs (18 kinds of *O,O*-dimethyl OPs and 6 kinds of *O,O*-diethyl OPs) used for the selectivity and sensitivity study are shown in Fig. 2. The results of selectivity and sensitivity determined by the optimized dcELISA based on mAb3C9 and hapten **6**-OVA are shown in Table 2. The results demonstrated that the developed dcELISA showed high sensitivity to 18 kinds of *O,O*-dimethyl OPs, with the IC_50_ in the range of 1.3–231.0 ng/mL, and the limits of detection (LOD, IC_10_) were determined as 0.3–52.4 ng/mL. However, for the 6 kinds of *O,O*-diethyl OPs, only azinphos-ethyl could be sensitively detected with IC_50_ of 95.3 ng/mL, the IC_50_ values of other *O,O*-diethyl OPs were determined as 1812.4–2824.3 ng/mL. The results indicated that mAb3C9 based dcELISA was class-specific for *O,O*-dimethyl OPs. Comparation of the IC_50_ values of OPs determined by the present study and the previous studies (Supplementary Table S2) showed that the dcELISA developed here exhibited the broadest selectivity and the highest sensitivity for *O,O*-dimethyl OPs. The OPs which were difficult to monitor in the previous studies, such as azinphos-methyl, chlorpyrifos-methyl, famphur, phosmet and tolclofos-methyl, could be sensitively detected in the present study.

The other mAb (mAb4D11) showed quite different selectivity for OPs compared to mAb3C9. The results of the selectivity and sensitivity study determined by the optimized dcELISAs based on mAb4D11 are shown in Supplementary Table S3. The results showed that only 9 kinds of *O,O*-dimethyl OPs could be sensitively detected by the dcELISA with IC_50_ in the range of 4.8–124.6 ng/mL. Moreover, 5 kinds of *O,O*-diethyl OPs including parathion, quinalphos, phoxim, triazophos and diazinon could be detected by mAb4D11-based dcELISA with IC_50_ in the range of 163.6–288.3 ng/mL. The mAb4D11-based dcELISA exhibited broad specificity for both *O,O*-diethyl and *O,O*-dimethyl OPs, but the selectivity for *O,O*-dimethyl OPs was less than that of mAb3C9-based dcELISA. Therefore, mAb3C9 was selected as the antibody used for *O,O*-dimethyl OPs class-specific monitoring.

### Selection of OP mimotopes from phage-display library

The chemicals used in chemosynthesis may be toxic to users, and the work required considerable effort^12^,^19^. To overcome this problem, peptide mimotopes that mimic the antibody binding site of low molecular weight compound can be used as competitors. Phage-display has proved to be a powerful tool to obtain mimotopes by panning against antibodies^12^,^21^,^22^,^23^. In this study, three rounds of panning against mAb3C9 were performed to enrich the positive phages from a commercial loop-constrained heptapeptide library. During the three rounds of panning, the concentration of coated mAb and parathion-methyl in elution buffer was successively reduced, but the output of phages was enriched from 1.2 × 10^5 ^pfu to 2.2 × 10^7 ^pfu. Forty-eight individual phages, which were randomly picked from the third round of panning (mimotopes named M1 to M48), were screened by phage ciELISA against parathion-methyl. The results indicated that only one mimotope was not responsive, and eight mimotopes showed no competition with 50 ng/mL parathion-methyl. The remaining thirty-nine mimotopes showed more or less competition with 50 ng/mL parathion-methyl. Fifteen mimotopes with higher sensitivity to parathion-methyl were selected for DNA sequencing, and the results demonstrated that five different mimotopes were obtained (sequences showed in Supplementary Table S4). Three of the five sequences held a core motif C-X-G-X-X-P-F-X-C (X represents a random amino acid), and M20 (C-T-G-T-T-P-F-Y-C), which exhibited superior sensitivity relative to the other mimotopes, was used for further study.

### Preparation of mimotope fusion protein

**Figure 3:**
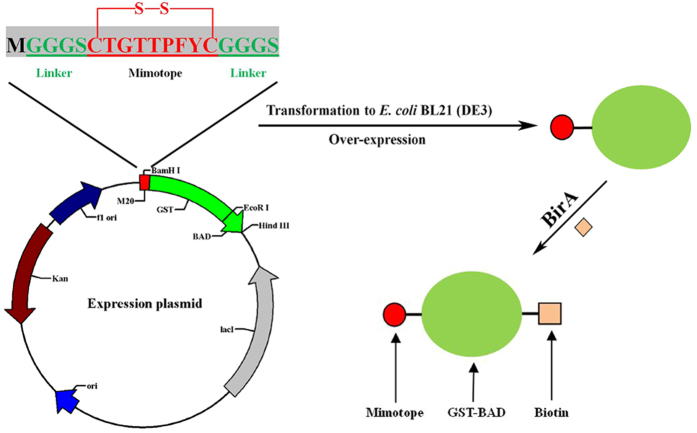
Schematic presentation of the expression and biotinylation of mimotope-GST-BAD fusion protein.

**Figure 4:**
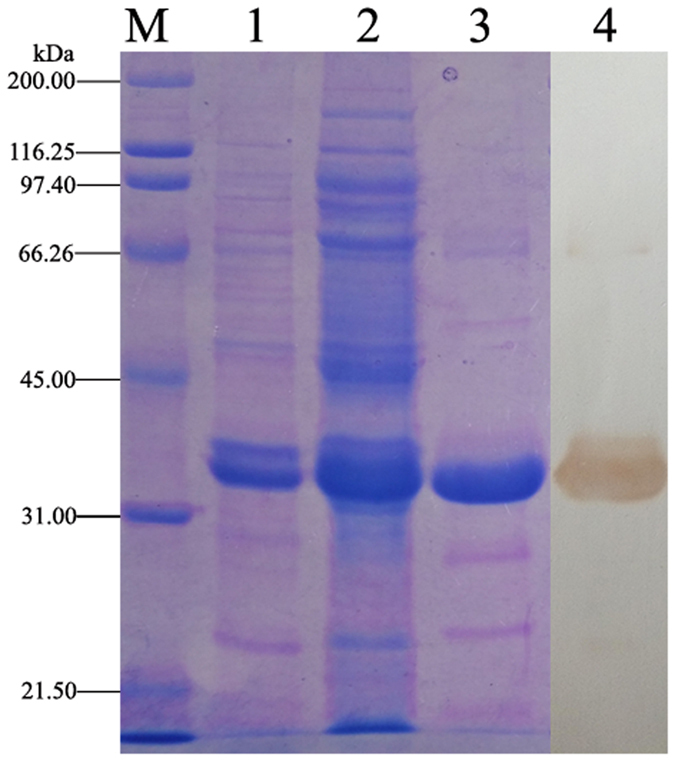
Characterization of the purified and biotinylated M20-GST-BAD fusion protein by SDS-PAGE and western blotting. Lane M: protein standards, lane 1: cell lysate pellet, lane 2: cell lysate supernatant, lane 3: purified M20-GST-BAD fusion protein (biotinylated), lane 4: western blotting of the biotinylated M20-GST-BAD fusion protein (detected by SA-HRP).

The gene fragment of M20 was synthesized as a part of the primer and fused with glutathione S-transferases (GST) coding gene by PCR. The M20-GST gene fragment was inserted into pET-28-BAD (a reconstructive pET-28 plasmid, including a biotin acceptor domain (BAD) composed of IgA hinge, Avi-tag and His-tag^28^) and expressed as M20-GST-BAD fusion protein. The BAD was added to allow the fusion protein to be site-specifically biotinylated by *E. coil* biotin ligase (BirA). The schematic diagram of the construction of the expression plasmid for M20-GST-BAD fusion protein is shown in Fig. 3. The fusion protein was soluble expressed in *E. coli* BL21(DE3) with high efficiency after only 6 h of induction with 0.5 mM IPTG at 30 °C (Fig. 4, lane 2). Moreover, the BirA was partially expressed as soluble protein (Supplementary Fig. S4) and the soluble BirA without His-tag in the supernatant could be used directly for *in vitro* biotinylation of M20-GST-BAD and removed by the following affinity purification with Ni-IDA resin (GenScript, Nanjing, Jiangsu, China). For *in vitro* biotinylation, the amount of BirA enzyme, D-biotin and ATP were assessed to confirm the complete biotinylation of the fusion protein. After biotinylation and purification, a total of 9.2 mg purified biotinylated fusion protein was obtained from the 100 mL-induced cell culture. The expression condition of the fusion protein was not further optimized because its yield was adequate for application in immunoassay. The biotinylated M20-GST-BAD was confirmed by SDS-PAGE and western blotting. As shown in Fig. 4 (lane 3), the molecular mass of the fusion protein is consistent with the theoretical value (calculated as 33 kDa for the 1:1:1 fusion of mimotope peptide, GST, and BAD). The western blotting result indicated that the fusion protein could be detected by streptavidin-HRP conjugate (SA-HRP) and confirmed the successful biotinylation of the fusion protein (Fig. 4, lane 4).

### Development of an innovative mimotope-based dcELISA

**Figure 5:**
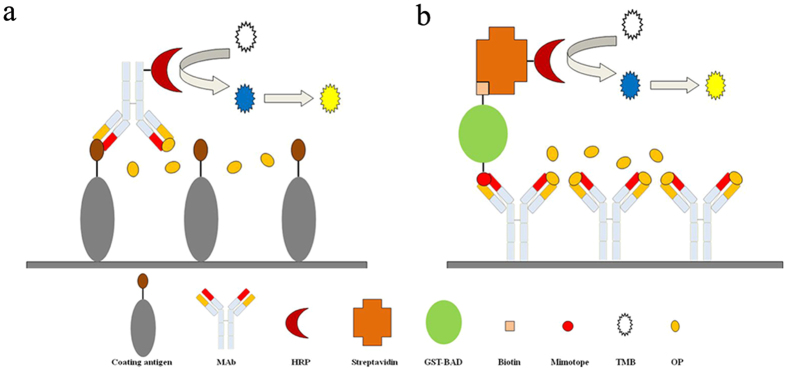
Schematic presentation of the developed chemosynthesized antigen-based dcELISA (**a**) and mimotope-based dcELISA (**b**).

To develop an easier mimotope-based dcELISA for *O,O*-dimethyl OPs detection, the biotinylated mimotope fusion protein (M20-GST-BAD) was marked with HRP for use as a competitor. The method only involved mixing the fusion protein with SA-HRP, and the mimotope fusion protein was marked with HRP owing to the high affinity between biotin and streptavidin. The mole ratios of biotinylated fusion protein and SA-HRP (the mole ratio of streptavidin and HRP is 1:1) were also optimized in this study. The results indicated that a low ratio (1:1) led to low affinity between the competitor and mAb3C9, whereas a high ratio (4:1) resulted in very high affinity but reduced the sensitivity of the developed immunoassay. The middle ratio (2:1) led to both high affinity and sensitivity and was thus selected as the optimum mole ratio (data not shown). The schematic of chemosynthesized antigen and mimotope-based dcELISAs is shown in Fig. 5. The ELISA conditions (ionic strength, pH and concentration of MeOH) were optimized as chemosynthesized antigen-based dcELISA, and the results showed no difference between the immunoassays. Compared to the previous ciELISA based on the biotinylated mimotope peptide, the mimotope-based dcELISA developed here showed its superiority by its shortened assay time and controllable ratio of mimotope and SA-HRP (the ratio could influence the sensitivity of the immunoassay)^26^.

**Figure 6:**
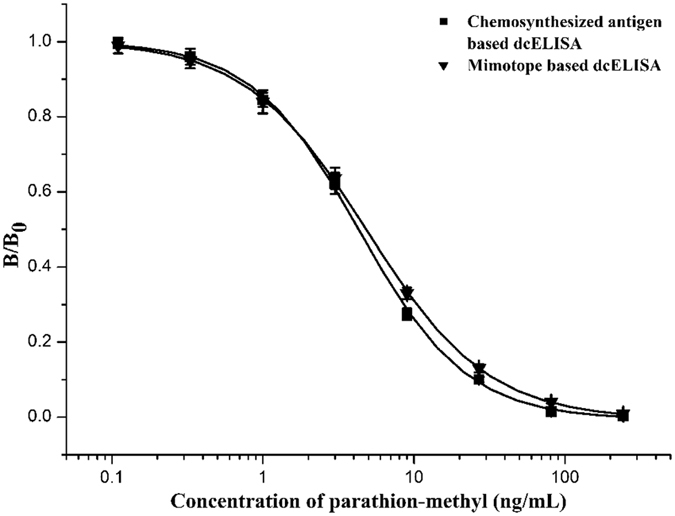
Standard curves of chemosynthesized antigen and mimotope-based dcELISAs for parathion-methyl. Each point represents the average of three replicates.

The selectivity and sensitivity of the developed mimotope-based dcELISA were determined. As shown in Table 2, the mimotope-based dcELISA showed sensitivity to 18 *O,O*-dimethyl OPs with IC_50_ ranging from 1.5 to 294.9 ng/mL, and the LOD values were determined as 0.4–63.2 ng/mL. The standard sigmoidal inhibition curves of chemosynthesized antigen and mimotope-based dcELISAs against parathion-methyl are highly similar and are shown in Fig. 6. Compared to the chemosynthesis of haptens, the screening of the mimotope is more convenient and eco-friendly.

### Pretreatment of samples

The matrix effect of food samples is always a major challenge for the application of immunoassay techniques. The common method to minimize matrix interference is dilution, but this method results in a significant reduction of sensitivity^7^. The traditional SPE (solid-phase extraction) pretreatment approach, including pretreatment of the sorbent, cleanup, elution and solvent evaporation steps, is time-consuming, labor-intensive, expensive and wasteful^29^. As an alternative, the QuEChERS (Quick, Easy, Cheap, Effective, Rugged, and Safe) method based on dispersive-SPE (d-SPE) is being widely employed in the pretreatment of food samples^30^,^31^. This pretreatment method involves only three steps: extraction of sample with organic solvent, transfer of the extract to a d-SPE tube and cleanup by d-SPE. The d-SPE uses less sorbent and equipment while also saving time, effort and money, thus making it more applicable in the sample preparation procedure before analysis by immunoassay^32^,^33^,^34^.

**Figure 7:**
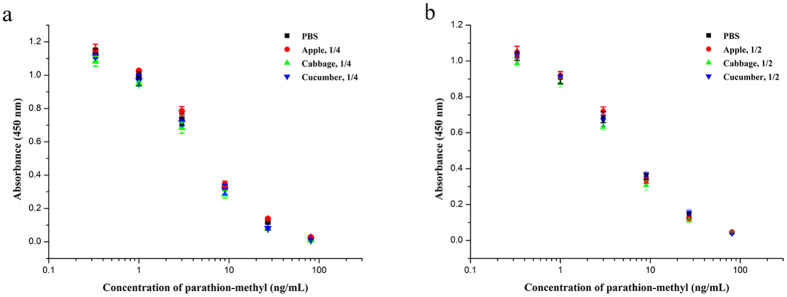
Matrix effects of d-SPE purified sample extracts. Samples were determined by chemosynthesized antigen-based dcELISA (**a**) and mimotope-based dcELISA (**b**). Sample 1/4 or 1/2 denotes that the sample extracts were diluted four or two times before analysis. Each point represents the average of three replicates.

The matrix effects of unpurified and d-SPE purified samples on chemosynthesized antigen and mimotope-based dcELISAs are compared in Supplementary Fig. S5. The extracts of apple, cabbage and cucumber without purification by d-SPE were diluted 5, 10 and 20 times before dcELISAs analysis. The results showed that matrix effects were decreased along with the dilution. However, more than 20 times dilution for chemosynthesized antigen-based dcELISA and 10 times dilution for mimotope-based dcELISA were needed to eliminate the matrix effects. In contrast, with d-SPE pretreatment, only 4 times dilution for chemosynthesized antigen-based dcELISA and 2 times dilution for mimotope-based dcELISA were needed. Compared with the direct dilution method, the additional steps of d-SPE lasting only a few minutes was sufficient to greatly reduce the matrix effects. To verify the validity of d-SPE pretreatment, the matrix effects of the d-SPE purified sample extracts, which contain different concentrations of parathion-methyl, were determined and compared with control (PBS). For chemosynthesized antigen-based dcELISA, as shown in Fig. 7a, 4 times dilution of d-SPE purified sample extracts was adequate to eliminate the interference caused by the matrix effects. In addition, for mimotope-based dcELISA, 2 times dilution was sufficient (Fig. 7b). The results indicated that a combination of QuEChERS and immunoassay was feasible and the mimotope-based dcELISA was more sensitive in sample analysis because of the less dilution.

### Validation of the chemosynthesized antigen and mimotope-based ELISAs

The practicability of the developed dcELISAs was confirmed by spike-recovery study. All d-SPE purified sample extracts were diluted 4 times to eliminate the matrix effects before analysis by the two dcELISAs. The recoveries of parathion-methyl, fenitrothion and azinphos-methyl that spiked in apple, cabbage and cucumber samples were calculated. The coefficient of variation (CV) was evaluated with three replicates. The results are shown in Supplementary Table S5 (for chemosynthesized antigen-based dcELISA) and Supplementary Table S6 (for mimotope-based dcELISA). For chemosynthesized antigen-based dcELISA, the recoveries of OPs in spiked samples were determined in the range of 90.3–112.8% with CV ranging from 3.5% to 14.1%. For mimotope-based dcELISA, recoveries were determined in the range of 92.4–121.5%, with CV ranging from 2.9% to 13.6%. Both of the developed dcELISAs showed satisfactory recoveries for OPs.

To evaluate the accuracy of the dcELISAs, a matrix-matched GC–MS/MS method that could analyze parathion-methyl, fenitrothion and azinphos-methyl in cucumber sample was also performed. The coefficient (*R*^*2*^) of the developed GC–MS/MS is 0.999 for parathion-methyl, 0.998 for fenitrothion and 0.998 for azinphos-methyl. For the correlation study of the dcELISAs (*X*) and GC–MS/MS (*Y*), the linear regression equation between chemosynthesized antigen-based dcELISA and GC–MS/MS was *Y *= 1.049*X* + 1.875, *R*^*2*^* *= 0.989 for parathion-methyl; *Y *= 1.036*X* + 2.629, *R*^*2*^* *= 0.995 for fenitrothion and *Y *= 0.923*X* + 1.937, *R*^*2*^* *= 0.998 for azinphos-methyl. The linear regression equations between mimotope-based dcELISA and GC–MS/MS was *Y *= 1.039*X* + 2.134, *R*^*2*^* *= 0.992 for parathion-methyl; *Y *= 1.067*X* − 1.732, *R*^*2*^* *= 0.997 for fenitrothion and *Y *= 0.963*X* − 0.926, *R*^*2*^* *= 0.997 for azinphos-methyl. The results indicated strong correlations between the developed dcELISAs and GC–MS/MS and indicated that the developed dcELISAs were reliable.

## Conclusions

In this study, mAb3C9 against the generic hapten of *O,O*-dimethyl OPs (hapten **1**, *O,O-dimethyl O-*(*3-carboxyphenyl*)*phosphorothioate*) was produced. To develop an effective class-specific immunoassay for *O,O*-dimethyl OPs, 20 chemosynthesized candidate coating antigens were prepared and optimized. With the optimized chemosynthesized coating antigen (Hapten **6**-OVA), a sensitive dcELISA was developed with the IC_50_ values of 18 *O,O*-dimethyl OPs ranging from 1.3 to 231.0 ng/mL. Compared with the trial and error of chemical synthesis, the goal-oriented, convenient and eco-friendly phage-display method to select mimotope of OPs would be more promising in the immunoassay. A mimotope of OPs (M20) was selected from a phage-display library by panning and fused with GST for over-expression as competitor. Based on the M20-GST, an innovative sensitive dcELISA was developed with the IC_50_ values of 18 *O,O*-dimethyl OPs ranging from 1.5 to 294.9 ng/mL. Additionally, both of the dcELISAs displayed satisfactory recoveries in samples analysis, and the mimotope-based dcELISA was more practical because a lower dilution ratio was needed to eliminate the matrix effects.

## Materials and Methods

### Preparation of haptens and hapten-protein conjugates

Twenty haptens were chemosynthesized and used in the present study. All of the haptens were synthesized using the same method as our previous study^17^. Briefly, the synthetic process includes three steps: I, esterification of the carboxyl group of the haptens’ spacer arm (hydroxybenzoic acid) in H_2_SO_4_/MeOH (1:10, v/v) solution; II, covalent coupling of the common structure of OPs (*O,O*-dimethyl or *O,O*-diethyl phosphorochlorodithioate) and the hydroxy of the haptens’ spacer arm (hydroxybenzoate); and III, hydrolysis of methyl benzoate of *O,O*-dimethyl or *O,O*-diethyl *O*-[(4-methyloxycarbonyl)phenyl]-phosphorothioate in absolute ethanol:1 M KOH (3:2) solution. The products were characterized by DRX-600 NMR spectroscopy (Bruker, Germany-Switzerland). The haptens were covalently attached to BSA or OVA using the active ester method to obtain hapten-protein conjugates^35^.

### Production of monoclonal antibody (mAb)

The preparation procedure of mAb is described as follows. Five 8-week-old female BALB/c mice were immunized intraperitoneally with 1:1 mixture (v/v) of hapten **1**-BSA (0.1 mg) and complete Freund’s adjuvant (200 μL of the mixture per mouse). Immunizations were repeated two times with incomplete Freund’s adjuvant at two-week intervals. One week after the last booster immunization, the serum of each mouse was collected from the caudal vein and determined by noncompetitive ELISA using both the homologous and heterologous coating antigens (hapten **1**-OVA and hapten **5**-OVA). Subsequently, the antisera were tested against parathion-methyl by ciELISA using the two coating antigens. The mouse with the highest titer was selected and euthanized for cell fusion. The splenocytes from the selected mouse were mixed with mouse myeloma cells (P3-X63-Ag8.653) at a 10:1 ratio and fused in the presence of 50% (w/v) PEG 4000 under 37 °C water bath. The fused cells were centrifuged, re-suspended with DMEM medium including 20% fetal bovine serum and added into four 96-well plates (50 μL/well). Next day, 50 μL/well of DMEM medium including 20% fetal bovine serum and HAT was added. Then, half of the medium in the wells was replaced by fresh HAT medium every two day. After 12 days, the culture supernatants of hybridomas were screened by ciELISA (hapten **5**-OVA was used as coating antigen and parathion-methyl was used as an analyte). The selected hybridomas were verified by ciELISA against chlorpyrifos-methyl, fenthion and fenitrothion. The selected positive hybridoma cell lines were transferred to a 24-well microculture plate in HT medium. Subsequently, the hybridoma cell lines were subcloned three times by limited dilution technique, and stable antibody-producing clones were expanded. The monoclonal hybridoma cells were injected intraperitoneally into the mice that had previously received an i.p. injection of 0.5 mL of pristane 1 week beforehand (10^6^ cells per mouse). Ten days later, the ascites fluids were collected and purified by ammonium sulfate. All animal procedures involving the care and use of animals were practiced in accordance with the ethics regulations of science research in the Institute of the Supervision, Inspection and Testing Center of Genetically Modified Organisms, Ministry of Agriculture (Beijing, China) and were approved by the Animal Experimental Welfare & Ethical Inspection Committee (No. 100034).

### Procedure of chemosynthesized antigen-based ELISA

The mAbs were labeled with HRP using modified NaIO_4_ method described by Tussen and Kurstak^36^. Briefly, 5 mg HRP was dissolved in 500 μL NaHCO_3_ (0.1 mol/L) solution and then 500 μL NaIO_4_ (16 mmol/L) solution was added. The mixture was stirred for 2 h at room temperature. After that, 2 mL of 7.5 mg/mL purified mAb that had been dialyzed against NaHCO_3_ buffer (0.1 mol/L, pH 9.5) was added and stirred for 3 h. After the reaction, 150 μL NaBH_4_ (5 mg/mL in 0.1 mmol/L NaOH) was added and stirred for 30 min at 4 °C. Then, another 450 μL fresh NaBH_4_ was added and stirred for 1 h at 4 °C. Finally, ammonium sulfate was added to reach a saturation concentration of 50% and the solution was stirred for 20 min in ice-water bath. The mixture was centrifuged at 10000 rpm for 10 min at 4 °C, and the precipitate was dissolved in 2 mL 1 × PBS (pH 7.4) to obtain mAb-HRP solution. The mAb-HRP solution was mixed with 2 mL glycerol and stored at −20 °C.

The dcELISA procedure was performed as below. First, the plate (Costar, Corning Inc., New York, USA) was coated with coating antigen (100 μL/well in 1 × PBS) for 1 h at 37 °C. Then, the wells were washed four times with PBST (1 × PBS with 0.05% (v/v) Tween-20) and blocked with 2% skim milk (200 μL/well) for 1 h at 37 °C. After washing four times, 50 μL analyte in MeOH-water solution and 50 μL mAb-HRP in PBS were added. The plate was incubated for 1 h at 37 °C and then washed four times before adding TMB solution (100 μL/well). After incubation for 15 min at 37 °C, 50 μL/well of H_2_SO_4_ (2 mol/L) was added to stop the reaction, and the absorbance was recorded at 450 nm using the Model 680 plate reader (Bio-Rad, USA).

### Optimization of coating antigen and dcELISA condition

To select the optimum coating antigen, all of the 20 hapten-OVA conjugates were tested for plate coating. Calibration curves of parathion-methyl were determined and fitted with a four-parameter logistic equation by Origin 7.0 (OriginLab, Northampton, MA, U.S.). The 50% inhibition values (IC_50_) of parathion-methyl were calculated. The ratio of maximal absorbance (*A*_max_) to IC_50_ value was used as a criterion. The coating antigen which obtained the highest ratio of *A*_max_/IC_50_ value was selected as the optimum antigen. Subsequently, the optimal condition for the dcELISA, such as the ionic strength, pH and concentration of methanol, was also determined. The highest ratio of *A*_max_/IC_50_ value was chosen as the optimum condition.

### Selectivity and sensitivity of chemosynthesized antigen-based dcELISA

18 kinds of *O,O*-dimethyl OPs and 6 kinds of *O,O*-diethyl OPs were purchased from National Standards of China and used for the selectivity and sensitivity study using the optimized dcELISA procedure. Calibration curves of OPs were determined and fitted with a four-parameter logistic equation by Origin 7.0. The IC_50_ value and detection limit of each OP were calculated. The CR value, which was used to evaluate the selectivity of the method, was calculated using the following equation:


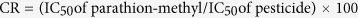


### Selection of OP mimotopes from the phage-display library

OP mimotopes were selected from a commercial loop-constrained heptapeptide library. Panning was performed according to the manufacturer’s instructions with some modification. Briefly, 1 mL of mAb was first coated onto a 12-well plate. Then, 1 mL of the library (2.0 × 10^11 ^pfu/mL) was added and incubated for 60 min at room temperature. The plate was washed 10 times with PBST (10 mM PBS with 0.1% (v/v) Tween-20, pH 7.4) followed by 10 times additional washes with PBS (10 mM, pH 7.4). Finally, specific phages were eluted with 500 μL parathion-methyl solution for 30 min. The concentrations of mAb used in the first, second and third rounds were 100, 10, and 1 μg/mL, respectively. And the concentrations of parathion-methyl were 1000, 100, and 10 ng/mL, respectively. A total of 48 individual phages from the third round were evaluated by competitive phage-ELISA method as described in previous study^12^.

### Preparation of biotinylated mimotope fusion protein

A plasmid pET-28-BAD was first reconstructed from pET-28a(+) vector by inserting BAD gene into pET-28a(+) between *Eco*R I and *Hin*d III sites. Mimotope gene fragment was designed in primer and fused with GST by PCR (M20-F: 5′-CGGATCC**TGTACGGGGACTACTCCGTTTTATTGC**GGTGGAGGTTCGATGTCCCCTATACTAG-3′ and GST-R: 5′-GGAATTCAGAGTCTGGCATGCTGTC-3′, the mimotope fragment is shown in blod type, the restriction sites are underlined) using GST encoding gene as template. Subsequently, the Mimotope-GST gene fragment was digested and inserted into pET-28-BAD. The prepared plasmid was transformed to *E. coli* BL21 (DE3) cells, screened by kanamycin and verified by individual bacterial colony PCR. The positive clone was inoculated into 10 mL LB broth (containing 50 μg/mL kanamycin) and shaken overnight at 37 °C (200 rpm). Next day, the culture was transferred into 100 mL LB broth (containing 50 μg/mL kanamycin) and shaken at 37 °C (200 rpm) for 1 h. Then, 500 μL of 0.1 M isopropylthio-*β*-D-galactoside (IPTG) was added and induced at 30 °C for 6 h (200 rpm). Subsequently, the cells were harvested, washed three times with buffer A (10 mM Tris-HCl, 20 mM NaCl, pH 8.0), suspended in 20 mL buffer A. The cells were broken by a ultrasonic processor and centrifuged at 12,000 rpm for 15 min at 4 °C to separate soluble fusion protein in the supernatant. The soluble fusion protein was transferred into a 50 mL tube contains 4 mL of buffer B (1 mM D-biotin, 100 mM ATP, pH 8.0) and 1 mL of the unpurified BirA enzyme (produced according to the following method). The mixture was incubated for 3 hours at room temperature and stored at 4 °C for 12 h to complete the biotinylation reaction. Finally, the mimotope fusion protein was purified by Ni-IDA resin according to the manufacturer’s instructions.

BirA enzyme was prepared by the following procedure. The gene encoding BirA was amplified by PCR with primer 1 (5′-CCCATGGGCATGAAGGATAACACCG-3′) and primer 2 (5′-CCAAGCTT**TTA** TTTTTCTGCACTACGC-3′) using pBirAcm plasmid as a template. After digestion with *Nco* I and *Hin*d III, the BirA gene was inserted into pET-28a(+) and transformed into *E. coli* BL21 (DE3). A stop codon (TAA) was added at the end of the *birA* gene to make sure that the enzyme was expressed without His-tag. The expression and separation steps of soluble BirA are the same as the above description. The supernatant (containing unpurified BirA enzyme) was used for *in vitro* biotinylation of the mimotope fusion protein directly.

### Development of mimotope-based dcELISA

To develop a mimotope-based dcELISA, the biotinylated mimotope fusion protein was firstly assembled with streptavidin-HRP conjugate (SA-HRP) utilized the high affinity of biotin and streptavidin. The assembly process was just mixing a certain amount of biotinylated mimotope fusion protein with SA-HRP and incubating them at 4 °C overnight before use as an HRP-labeled competitor.

An innovative mimotope-based dcELISA was developed and performed as below. The plate was coated (100 μL/well) with mAb for 1 h at 37 °C and blocked with 2% skim milk (200 μL/well). After washing four times by PBST, 50 μL analyte in MeOH-water solution and 50 μL HRP-labeled mimotope in PBS were added. The plate was incubated for 1 h at 37 °C and washed four times. TMB solution (100 μL/well) was added and incubated for 15 min at 37 °C. Finally, 2 mol/L H_2_SO_4_ (50 μL/well) was added to stop the reaction, and the absorbance was recorded at 450 nm.

The optimal condition for mimotope-based dcELISA was optimized as chemosynthesized antigen-based dcELISA. The selectivity and sensitivity of the developed mimotope-based dcELISA for the 24 OPs were also determined under the optimum condition.

### Preparation of samples

The QuEChERS (Quick, Easy, Cheap, Effective, Rugged, and Safe) approach based on dispersive solid-phase extraction (d-SPE) was employed to prepare the samples, including apple, cucumber and cabbage. The preparation procedure was as follows. A sample was washed and homogenized by a homogenizer, and 10 g of the homogenized sample was placed in a 50-mL polypropylene tube. After addition of 10 mL MeCN, the tube was vortexed for 1 min. Subsequently, 1 g NaCl and 4 g MgSO_4_ (anhydrous) were added to the tube, followed by another 1 min of shaking. Then, the tube was centrifuged at 6,000 rpm for 5 min and 1 mL extract was transferred to a d-SPE tube. The 2 mL d-SPE tube contained 150 mg MgSO_4_ (anhydrous) and 100 mg PSA (Bondesil-primary secondary amine, Silibase, China). The tube was capped and shaken for 30 s followed by centrifugation for 3 min at 10,000 rpm. Then, 500 μL of the supernatant was transferred into a glass tube and dried under a stream of nitrogen at 40 °C. The residue was redissolved with MeOH-water before dcELISA analyses.

For GC–MS/MS analysis, samples were pretreated using the QuEChERS method as described in our previous study^17^.

### Validation of the developed dcELISAs

For the spike-recovery study, 3 *O,O*-dimethyl OPs (parathion-methyl, fenitrothion and azinphos-methyl) were spiked to OPs-free samples with known amounts (each sample contained one pesticide). Then, the samples were thoroughly mixed and incubated for 1 h before extraction and purification by QuEChERS. The residues were analyzed by the optimum dcELISAs, and the OP concentration was calculated using the calibration curves.

The correlation study of dcELISAs and GC–MS/MS was performed as follows. Cucumber samples spiked with OPs (25, 50 and 100 ng/g) were analyzed by dcELISAs and GC–MS/MS. The GC–MS/MS analysis of *O,O*-dimethyl OPs (parathion-methyl, fenitrothion and azinphos-methyl) in the spiked cucumber samples was developed using a Shimadzu GC/MS-TQ8030 spectrometer (Scan/MRM mode). The calibration curves were evaluated with matrix-matched standard calibrations in blank extracts of cucumber (five concentrations including 20, 50, 100, 200, and 400 ng/mL were used). The GC separation was performed on an Rtx-5 MS column (30 m × 0.25 mm × 0.25 μm film thickness). The Electron ionization mode at an ionizing energy of 70 eV was used with the ion source at 230 °C. The OPs concentrations in the spiked cucumber samples were determined and the linear regression equations between ELISAs and GC–MS/MS were calculated.

## Additional Information

**How to cite this article**: Zhao, F. *et al*. Development and comparative study of chemosynthesized antigen and mimotope-based immunoassays for class-specific analysis of *O,O*-dimethyl organophosphorus pesticides. *Sci. Rep.* **6**, 37640; doi: 10.1038/srep37640 (2016).

**Publisher’s note:** Springer Nature remains neutral with regard to jurisdictional claims in published maps and institutional affiliations.

## Supplementary Material

Supplementary Information

## Figures and Tables

**Table 1:** Selection of the optimum combinations of mAbs and coating antigens of dcELISAs.

Coating antigen	mAb3C9			mAb4D11			
	*A*_max_	IC_50_^a^	*A*_max_/IC_50_ (%)	*A*_max_	IC_50_^a^	*A*_max_/IC_50_ (%)	
Hapten **1**-OVA	1.83	127.3	1.4	1.69	232.5	0.7	
Hapten **2**-OVA	1.61	45.1	3.6	1.92	423.4	0.5	
Hapten **3**-OVA	1.67	118.9	1.4	1.72	104.7	1.6	
Hapten **4**-OVA	1.56	38.5	4.0	2.03	265.7	0.8	
Hapten **5**-OVA	1.56	16.6	9.4	1.88	63.8	2.9	
Hapten **6**-OVA	**1.39**	**5.2**	**26.7**	1.84	87.0	2.1	
Hapten **7**-OVA	1.14	50.5	2.3	**1.70**	**22.1**	**7.7**	
Hapten **8**-OVA	1.44	13.2	10.9	1.71	27.5	6.2	
Hapten **9**-OVA	1.56	85.0	1.8	1.94	63.5	3.1	
Hapten **10**-OVA	1.61	18.9	8.5	1.85	70.2	2.6	
Hapten **11**-OVA	1.63	56.7	2.9	1.67	65.9	2.5	
Hapten **12**-OVA	1.46	14.9	9.8	1.69	73.9	2.3	
Hapten **13**-OVA	1.21	41.5	2.9	1.93	115.7	1.7	
Hapten **14**-OVA	1.65	18.8	8.8	1.91	163.2	1.2	
Hapten **15**-OVA	1.73	103.5	1.7	2.03	302.4	0.7	
Hapten **16**-OVA	1.73	44.1	3.9	2.02	322.5	0.6	
Hapten **17**-OVA	1.62	38.9	4.2	1.86	118.3	1.6	
Hapten **18**-OVA	1.56	13.1	11.9	2.06	106.1	1.9	
Hapten **19**-OVA	0.53	12.1	4.4	0.78	11.1	7.0	
Hapten **20**-OVA	0.61	11.4	5.4	0.96	12.8	7.5	

^a^IC_50_ values are in units of ng/mL.

**Table 2:** Comparation of sensitivity and selectivity of the chemosynthesized antigen and mimotope-based dcELISAs for OPs.

No.	Analytes	Hapten **6**-OVA			M20-GST-BAD		
		IC_50_^a^ ± SD	CR (%)^b^	LOD^a^	IC_50_^a^ ± SD	CR (%)^b^	LOD^a^
1	Parathion-methyl^c^	4.7 ± 0.3	100.0	0.9	5.1 ± 0.2	100.0	0.9
2	Famphur^c^	1.3 ± 0.1	347.8	0.3	1.5 ± 0.1	339.6	0.4
3	Phosmet^c^	1.4 ± 0.1	323.6	0.3	1.5 ± 0.1	332.9	0.3
4	Azinphos-methyl^c^	1.5 ± 0.1	306.6	0.4	1.7 ± 0.1	290.8	0.4
5	Methidathion^c^	2.9 ± 0.1	162.9	0.6	3.2 ± 0.2	157.6	0.6
6	Cyanophos^c^	3.1 ± 0.2	152.8	0.6	3.5 ± 0.1	146.2	0.7
7	Chlorpyrifos-methyl^c^	3.9 ± 0.1	120.7	0.7	4.1 ± 0.2	122.8	0.9
8	Fenitrothion^c^	4.4 ± 0.3	107.1	1.1	4.9 ± 0.2	104.1	1.2
9	Tolclofos-methyl^c^	6.6 ± 0.3	70.2	1.2	8.3 ± 0.4	61.3	1.7
10	Fenthion^c^	14.5 ± 1.1	32.1	3.0	23.4 ± 1.7	21.6	4.4
11	Fenchlorphos^c^	44.3 ± 2.2	10.5	7.4	50.1 ± 2.1	10.1	9.3
12	Tetrachlorvinphos^c^	54.4 ± 2.1	8.6	8.9	59.7 ± 3.3	8.5	11.2
13	Bromophos-methyl^c^	75.5 ± 4.1	6.2	19.5	82.2 ± 2.4	6.2	19.4
14	Pirimiphos-methyl^c^	77.4 ± 3.3	6.0	15.6	83.7 ± 3.8	6.0	22.2
15	Azinphos-ethyl^d^	95.3 ± 4.2	4.9	21.6	107.4 ± 3.2	4.7	26.4
16	Dichlorvos^c^	132.1 ± 6.1	3.5	24.0	145.4 ± 3.8	3.5	31.2
17	Dimethoate^c^	170.3 ± 7.3	2.7	18.4	189.3 ± 8.1	2.7	28.4
18	Monocrotophos^c^	179.1 ± 8.6	2.6	43.2	201.4 ± 4.3	2.5	61.6
19	Paraoxon-methyl^c^	231.0 ± 12.1	2.0	52.4	294.9 ± 9.3	1.7	63.2
20	Diazinon^d^	1812.4 ± 46.1	0.3	391.7	2064.8 ± 41.7	0.2	412.3
21	Parathion^d^	2412.5 ± 61.0	0.2	466.3	2328.4 ± 51.4	0.2	439.2
22	Phoxim^d^	2652.4 ± 112.0	0.2	435.4	2577.9 ± 72.1	0.2	433.6
23	Triazophos^d^	2786.5 ± 67.7	0.2	482.4	2972.1 ± 73.3	0.2	474.2
24	Quinalphos^d^	2824.3 ± 95.2	0.2	512.3	3226.6 ± 59.2	0.2	531.8

^a^IC_50_ and LOD values are in units of ng/mL.

^b^CR (%) was calculated by the equation (IC_50_ of parathion-methyl/IC_50_ of pesticide) × 100.

^c^*O,O*-dimethyl OPs.

^d^*O,O*-diethyl OPs.
