# Supplementary material for: Development and comparative study of chemosynthesized antigen and mimotope-based immunoassays for class-specific analysis of O,O-dimethyl organophosphorus pesticides
Source: Sci Rep. 2016 Nov 22;6:37640. doi: 10.1038/srep37640 (PMC5118688; doi:10.1038/srep37640)
Supplement: Supplementary Information [file srep37640-s1.pdf]

Supplementary Information

Development and comparative study of chemosynthesized antigen and mimotope-based immunoassays for class-specific analysis of *O,O*-dimethyl organophosphorus pesticides

Fengchun Zhao, Huimin Wang, Xiao Han, and Zhengyou Yang*

Department of Microbiology, College of Life Science, Key Laboratory for Agriculture Microbiology, Shandong Agricultural University, Taian 271018, China.

* To whom correspondence should be addressed. Email: zhyouyang@sdau.edu.cn

Table S1. Yields and ^{1}H-NMR results of chemosynthesized haptens.

Hapten	Yield	^{1}H-NMR (600 MHz, $CDCl_3$)
Hapten **1**	39.10%	δ 7.99-7.95 (m, 1H), 7.91-7.89 (m, 1H), 7.50-7.43 (m, 2H), 3.96-3.78 (m, 6H)
Hapten **2**	71.20%	δ 7.98-7.94 (m, 1H), 7.92 (dd, J = 2.5, 1.8 Hz, 1H), 7.49-7.45 (m, 2H), 4.27 (dq, J = 10.0, 7.1 Hz, 4H), 1.39 (td, J = 7.1, 0.8 Hz, 6H)
Hapten **3**	59.20%	δ 8.13 (d, J = 8.6 Hz, 2H), 7.30-7.27 (m, 2H), 3.88 (d, J = 13.9 Hz, 6H)
Hapten **4**	78.10%	δ 8.12 (t, J = 5.5 Hz, 2H), 7.31-7.27 (m, 2H), 4.34-4.18 (m, 4H), 1.43-1.32 (m, 6H)
Hapten **5**	29.30%	δ 8.03 (d, J = 7.8 Hz, 1H), 7.59-7.52 (m, 1H), 7.34 (d, J = 8.3 Hz, 1H), 7.29 (t, J = 7.6 Hz,1H), 3.89 (d, J = 13.9 Hz, 6H)
Hapten **6**	48.10%	δ 8.02 (d, J = 7.8 Hz, 1H), 7.60-7.54 (m, 1H), 7.40 (d, J = 8.3 Hz, 1H), 7.29 (t,J = 7.6 Hz, 1H), 4.29 (dq, J = 9.5, 7.1 Hz, 4H), 1.37 (dt, J = 7.9, 7.1 Hz, 6H)
Hapten **7**	42.40%	δ 7.97 (d, J = 8.6 Hz, 1H), 7.89 (d, J = 1.7 Hz, 1H), 7.01 (d, J = 8.6 Hz, 1H), 4.00 – 3.85 (m, 9H)
Hapten **8**	72.50%	δ 7.96 (dt, J = 13.8, 6.9 Hz, 1H), 7.92 (t, J = 1.9 Hz, 1H), 7.00 (d, J = 8.6 Hz, 1H), 4.29 (dq, J = 9.6, 7.1 Hz, 4H), 3.93 (s, 3H), 1.40 (t, J = 7.1 Hz, 6H)
Hapten **9**	39.80%	δ 8.06-7.76 (m, 2H), 7.33 (d, J = 7.9 Hz, 1H), 3.90 (d, J = 13.9 Hz, 6H), 2.40 (s, 3H)
Hapten **10**	68.20%	δ 7.95 (s, 1H), 7.85 (d, J = 7.9 Hz, 1H), 7.32 (d, J = 7.9 Hz, 1H), 4.28 (dq, J = 9.9, 7.1 Hz,4H), 2.40 (s, 3H), 1.40 (td, J = 7.0, 0.8 Hz, 6H)
Hapten **11**	37.10%	δ 7.86 (d, J = 1.7 Hz, 1H), 7.32-7.28 (m, 1H), 7.27 (d, J = 3.6 Hz, 1H), 3.88 (d, J = 13.8 Hz,6H), 2.63 (s, 3H)
Hapten **12**	69.30%	δ 7.88 (s, 1H), 7.30 (dt, J = 9.8, 4.9 Hz, 1H), 7.26 (t, J = 4.1 Hz, 1H), 4.35-4.15 (m, 4H), 2.63(s, 3H), 1.46-1.32 (m, 6H)
Hapten **13**	52.10%	δ 7.74 (dd, J = 8.3, 1.5 Hz, 1H), 7.70 (d, J = 1.0 Hz, 1H), 7.29 (dd, J = 8.3, 1.7 Hz, 1H), 3.96-3.88 (m, 9H)
Hapten **14**	81.40%	δ 7.80-7.64 (m, 2H), 7.32 (dd, J = 8.3, 1.7 Hz, 1H), 4.29 (dq, J = 9.7, 7.1 Hz, 4H), 3.93 (s, 3H), 1.45-.33 (m, 6H)
Hapten **15**	49.70%	δ 7.99 (s, 1H), 7.95 (dd, J = 8.5, 1.9 Hz, 1H), 7.32 (dd, J = 8.5, 1.2 Hz, 1H), 3.89 (d, J = 13.9Hz, 6H), 2.37 (s, 3H)
Hapten **16**	79.40%	δ 7.98 (d, J = 6.0 Hz, 1H), 7.97-7.92 (m, 1H), 7.36 (d, J = 8.5 Hz, 1H), 4.27 (dq, J = 9.3, 7.1Hz, 4H), 2.37 (s, 3H), 1.39 (td, J = 7.1, 0.5 Hz, 6H)
Hapten **17**	48.20%	δ 8.09 (d, J = 8.6 Hz, 1H), 7.12-7.09 (m, 1H), 7.08 (s, 1H), 3.88 (d, J = 13.9 Hz, 6H), 2.66 (s, 3H)
Hapten **18**	75.60%	δ 8.10 (t, J = 7.6 Hz, 1H), 7.11 (d, J = 9.1 Hz, 1H), 7.09 (s, 1H), 4.34 -4.18 (m, 4H), 2.66 (s, 3H), 1.38 (td, J = 7.1, 0.6 Hz, 6H)
Hapten **19**	25.40%	δ 10.74 (s, 1H), 7.94 (d, J = 8.0 Hz, 1H), 7.14 (s, 1H), 7.10 (d, J = 8.1 Hz, 1H), 3.91 (d, J = 13.9 Hz, 6H), 2.42 (s, 3H)
Hapten **20**	37.80%	δ 7.91 (d, J = 8.0 Hz, 1H), 7.19 (s, 1H), 7.08 (d, J = 8.0 Hz, 1H), 4.38-4.19 (m, 4H), 2.41 (s, 3H), 1.38 (td, J = 7.1, 0.8 Hz, 6H)

Table S2. Comparision of the IC_{50} values (ng/mL) of immunoassays for *O,O*-dimethyl OPs developed in this study and in the literatures. [a]*O*,*O*-dimethyl OPs. [b]*O*,*O*-diethyl OPs. [c]OPs not used in this study.

No.	Analytes	This stduy (mAb3C9) 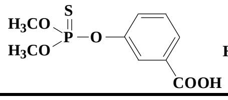	Hua et al.[11] 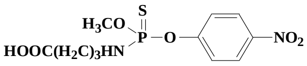	Liang et al.[9] 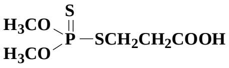	Liu et al.[10] 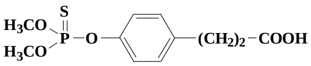
1	Parathion-methyl[a]	4.7	3.7	438.9	580
2	Famphur[a]	1.3	58215.7	-	-
3	Phosmet[a]	1.4	-	159.7	-
4	Azinphos-methyl[a]	1.5	>100000	-	-
5	Cyanophos[a]	2.9	20.3	-	-
6	Methidathion[a]	3.1	-	191.7	-
7	Chlorpyrifos-methyl[a]	3.9	>100000	-	810
8	Fenitrothion[a]	4.4	8.9	324	3540
9	Tolclofos-methyl[a]	6.6	>100000	-	1100
10	Fenthion[a]	14.5	>100000	788.9	1210
11	Fenchlorphos[a]	44.3	-	-	-
12	Tetrachlorvinphos[a]	54.4	-	-	-
13	Bromophos-methyl[a]	75.5	-	-	-
14	Pirimiphos-methyl[a]	77.4	-	-	-
15	Azinphos-ethyl[b]	95.3	-	-	-
16	Dichlorvos[a]	132.1	>100000	-	-
17	Dimethoate[a]	170.3	>100000	28.9	-
18	Monocrotophos[a]	179.1	-	-	-
19	Paraoxon-methyl[a]	231.0	118.7	-	-
20	Diazinon[b]	1812.4	-	-	-
21	Parathion[b]	2412.5	11.3	>1000	-
22	Phoxim[b]	2652.4	>100000	-	-
23	Triazophos[b]	2786.5	>100000	-	-
24	Quinalphos[b]	2824.3	-	-	-
25	EPN[b]	-[c]	18.3	-	-
26	Paraoxon-ethyl[b]	-	162.2	-	-
27	Malathion[a]	-	>100000	30.1	10470
28	Isochlorthion[a]	-	576.6	-	-

Table S3. Sensitivity and selectivity of the mAb4D11-based dcELISA for OPs. [a]IC_{50} values are in units of ng/mL. [b]CR (%) was calculated by the equation [(IC_{50} of parathion/IC_{50} of analyte)] × 100. [c]No detection.

No.	Analytes	IC_{50}[a]	CR(%)[b]	LOD[a]
1	Parathion-methyl	14.5	100.0	2.0
2	Fenthion	4.8	303.6	0.9
3	Fenitrothion	8.1	180.2	1.2
4	Cyanophos	17.6	82.5	2.3
5	Chlorpyrifos-methyl	24.4	59.4	5.7
6	Tolclofos-methyl	31.4	46.2	9.5
7	Fenchlorphos	55.0	26.4	13.3
8	Pirimiphos-methyl	62.1	23.4	9.1
9	Bromophos-methyl	124.6	11.6	31.4
10	Parathion	163.6	8.9	17.5
11	Quinalphos	184.2	7.9	32.5
12	Phoxim	244.1	5.9	49.5
13	Triazophos	279.7	5.2	59.9
14	Diazinon	288.3	5.0	73.2
15	Famphur	346.4	4.2	80.3
16	Dichlorvos	1827.9	0.8	437.2
17	Tetrachlorvinphos	2431.3	0.6	536.3
18	Paraoxon-methyl	2643.3	0.5	512.5
19	Monocrotophos	2914.5	0.5	594.3
20	Azinphos-methyl	>5000	<0.3	ND[c]
21	Azinphos-ethyl	>5000	<0.3	ND
22	Dimethoate	>5000	<0.3	ND
23	Phosmet	>5000	<0.3	ND
24	Methidathion	>5000	<0.3	ND

Table S4. Mimotope sequences and IC_{50} of parathion-methyl determined by phage-ELISA.

Mimotope name	Mimotope sequence	A_{max}	IC_{50} (ng/mL)
M3 (M7, M12)	C L Y H P W N N C	1.41	20.1
M4 (M8, M10, M12)	C L G T T P F F C	1.52	16.9
**M20 (M21, M25)**	**C T G T T P F Y C**	**1.64**	**12.6**
M32 (M35, M37)	C R G S M P F W C	1.38	18.4
M39 (M42)	C M A R Y M S A C	1.59	13.2

Table S5. Recoveries of OPs from spiked samples determined by the chemosynthesized antigen-based dcELISA (n=3). [a]The OPs in spiked samples are in unit of ng/g. [b]R is the average recovery.

Samples	Analyte	Azinphos-methyl		Fenitrothion		Parathion-methyl	
	Spiked[a]	5	10	10	20	10	20
Apple	R (%)	95.6	102.5	97.7	91.8	90.3	108.2
	CV (%)	4.5	7.6	10.2	8.9	14.1	8.2
Cabbage	R (%)	93.9	97.8	101.2	98.2	112.8	101.7
	CV (%)	13.2	3.5	9.3	6.8	5.5	4.8
cucumber	R (%)	101.7	108.5	101.8	94.7	106.2	101.3
	CV (%)	3.9	6.5	8.7	10.1	6.4	5.8

Table S6. Recoveries of OPs from spiked samples determined by the mimotope-based dcELISA (n=3). [a]The OPs in spiked samples are in unit of ng/g. [b]R is the average recovery.

Samples	Analyte	Azinphos-methyl		Fenitrothion		Parathion-methyl	
	Spiked[a]	5	10	10	20	10	20
Apple	R (%)	92.4	102.5	97.7	94.8	93.3	108.2
	CV (%)	4.7	4.9	6.6	9.8	6.7	4.3
Cabbage	R (%)	93.9	97.8	101.2	98.2	121.5	101.7
	CV (%)	10.4	6.2	13.6	7.7	3.8	5.6
cucumber	R (%)	101.7	108.5	101.8	94.7	106.2	101.3
	CV (%)	8.2	6.7	9.4	11.2	2.9	3.9

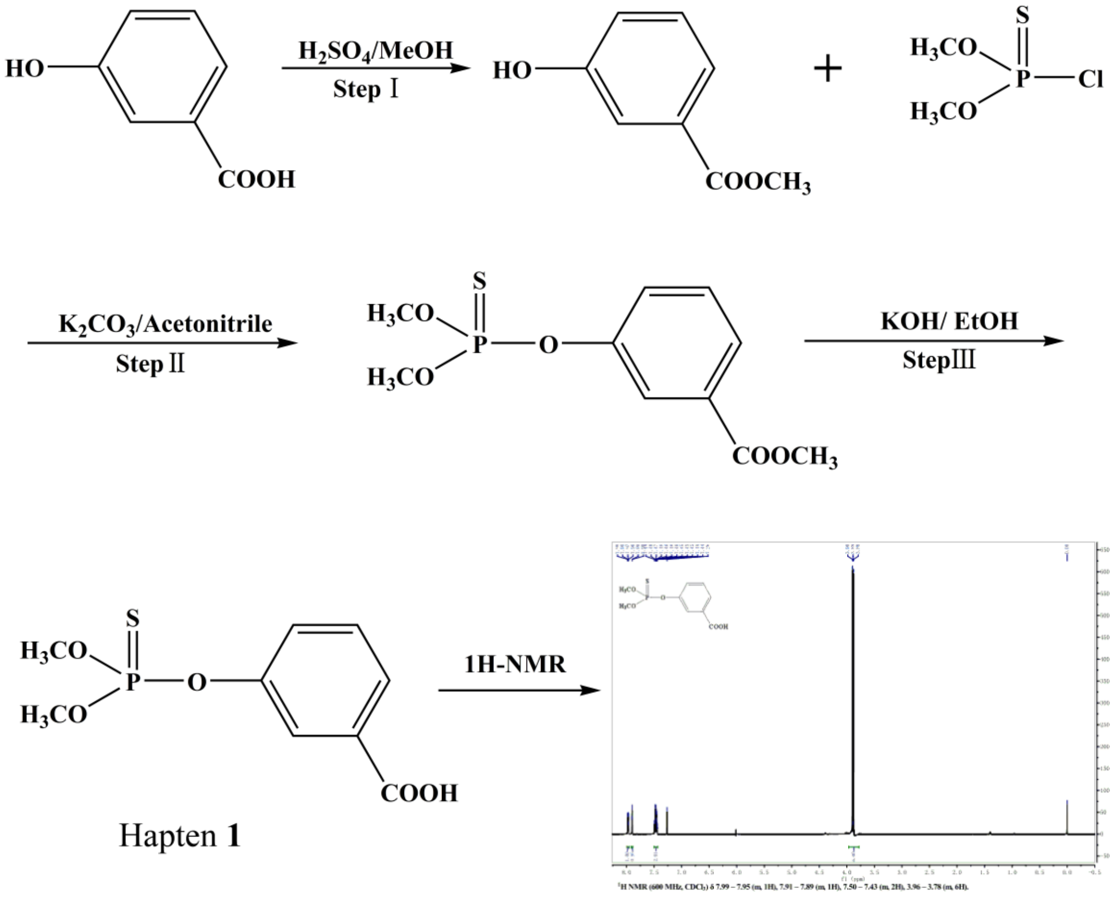

Figure S1. Synthetic route of hapten **1**.

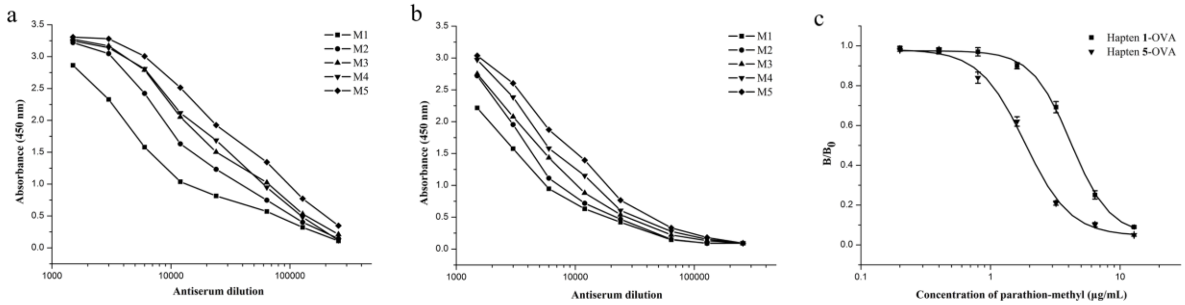

Figure S2. Detection of mice antisera after three times immunization. (**a**) Antisera titers determination using hapten **1**-OVA (10 μg/mL) as coating antigen. (**b**) antisera titers determination using hapten **5**-OVA (10 μg/mL) as coating antigen. (**c**) B/B_0 values of antiserum M5 against parathion-methyl.

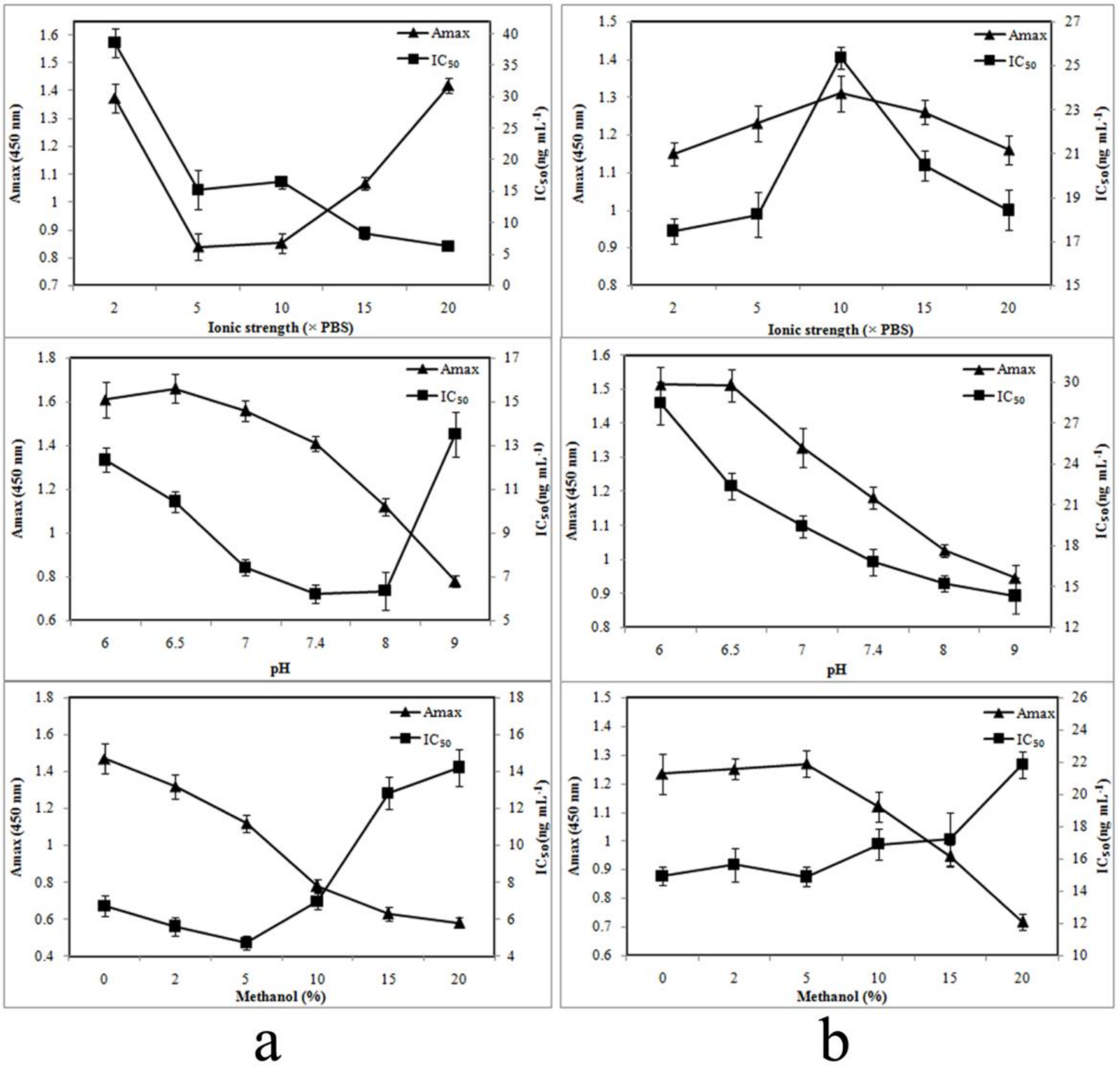

Figure S3. Effect of ionic strength, pH, and concentration of methanol on the dcELISAs based on mAb3C9 (a) and mAb4D11 (b). Parathion-methyl was used as analyte. Each point represents the average of three replicates.

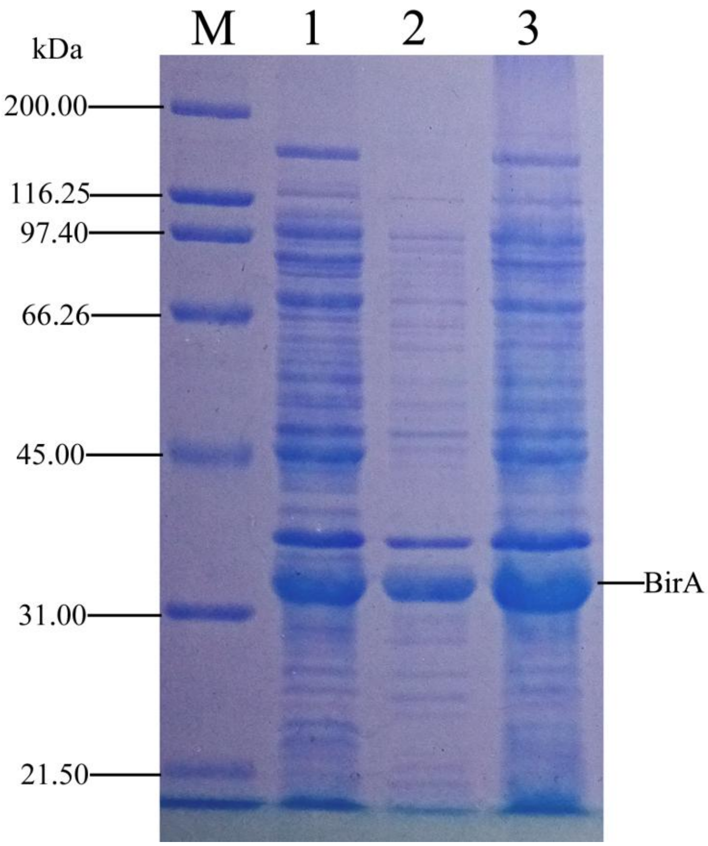

Figure S4. Characterization of BirA by SDS-PAGE. Lane M: protein standards, lane 1: cell lysate supernatant, lane 2: cell lysate pellet, lane 3: total cell lysate.

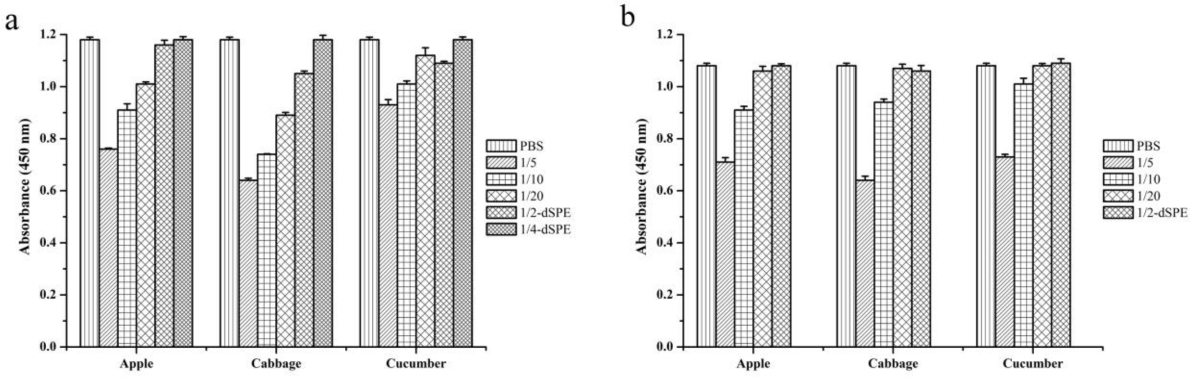

Figure S5. Matrix effects of purified and unpurified apple, cabbage and cucumber samples analyzed by chenmosynthesized antigen-based dcELISA (a) and mimotope-based dcELISA (b). PBS was used as control. Unpurified sample extracts were diluted 5, 10 and 20 times before dcELISA analyses. Sample extracts purified by dSPE-based QuEChERS were diluted 2 and 4 times before chemosynthesized antigen-based dcELISA analyses, and 2 times before mimotope-based dcELISA analyses(n=4).
